# Sciatic Nerve Plastic Surgery using Autologous Adipose Tissue

**DOI:** 10.17691/stm2023.15.1.04

**Published:** 2023-01-28

**Authors:** A.G. Velichanskaya, М.L. Bugrova, E.V. Pogadaeva, E.A. Ermolina, A.V. Yudintsev, I.L. Ermolin

**Affiliations:** Associate Professor, Department of Histology with Cytology and Embryology; Privolzhsky Research Medical University, 10/1 Minin and Pozharsky Square, Nizhny Novgorod, 603005, Russia;; Associate Professor, Head of the Department of Histology with Cytology and Embryology; Privolzhsky Research Medical University, 10/1 Minin and Pozharsky Square, Nizhny Novgorod, 603005, Russia;; Senior Laboratory Assistant, Department of Histology with Cytology and Embryology; Privolzhsky Research Medical University, 10/1 Minin and Pozharsky Square, Nizhny Novgorod, 603005, Russia;; Senior Lecturer, Department of Biology; Privolzhsky Research Medical University, 10/1 Minin and Pozharsky Square, Nizhny Novgorod, 603005, Russia;; Associate Professor, Department of Biophysics; National Research Lobachevsky State University of Nizhni Novgorod, 23 Prospekt Gagarina, Nizhny Novgorod, 603022, Russia Researcher; National Research Lobachevsky State University of Nizhni Novgorod, 23 Prospekt Gagarina, Nizhny Novgorod, 603022, Russia; Professor, Department of Histology with Cytology and Embryology; Privolzhsky Research Medical University, 10/1 Minin and Pozharsky Square, Nizhny Novgorod, 603005, Russia;

**Keywords:** sciatic nerve regeneration, sciatic nerve plastic surgery, autologous adipose tissue

## Abstract

**The aim of the investigation** is to study structural alterations of autologous omental adipose tissue in a silicon conduit and to evaluate its possible use for regeneration of the sciatic nerve in diastasis.

**Materials and Methods:**

Mature outbred male Wistar rats have been used in the study. The animals were divided into 7 experimental groups with complete transection of the sciatic nerve on the right side at the mid-third level of the thigh. The ends of the transected nerve were pulled apart, inserted into a silicon conduit, and secured to the epineurium. The conduit of group 1 (control) was filled with a saline solution; in group 2, it was filled with an autologous omental adipose tissue with saline solution. Intravital labeling of the omental adipose tissue with the lipophilic PKH 26 dye (in group 3) was used for the first time to find out whether the omental cells were involved in formation of the regenerating nerve. Diastasis in groups 1–3 was 5 mm, the postoperative period was 14 weeks. The dynamics of the omental adipose tissue changes in groups 4–7 was assessed by placing the omental tissues into the conduit covering 2 mm of diastasis. The postoperative period was 4, 14, 21, and 42 weeks.

**Results:**

In group 2 (omental adipose tissue + saline), the clinical condition of the damaged limb after 14 weeks may be evaluated as satisfactory and approximating to the intact parameters as compared to group 1 where the conduit was filled with a saline solution only. The sum of large and medium-sized nerve fibers in group 2 was 2.7 times greater than that in group 2. The milled omental adipose tissue inside the conduit changed its volume and structure in nerve diastasis and was constantly utilized up to complete elimination over time. The omental cells integrated into the newly formed nerve in the graft area.

**Conclusion:**

As a graft, the adipose tissue of the autologous omentum produces a stimulating effect on the post-traumatic regeneration of the sciatic nerve.

## Introduction

The problem of post-traumatic regeneration of the peripheral nerve in diastasis using its stump is one of the vital issues of neurosurgery [1, 2]. In medical practice, autografts of a healthy afferent cutaneous nerve are often used in large diastases, which in its turn results in sensitivity disturbances in the injured limb [3-6]. One of the current solutions of this problem is reparation of the injured nerve trunk with conduits (tubes) filled with different media which stimulate its regeneration [5-11]. Adipose tissue-derived stem cells (ADSCs) are most widely used as fillers owing to their unique characteristics. ADSCs have a phenotype and profiles of gene expression similar to mesenchymal stem cells of the bone marrow and have some advantages: a high percentage of multipotent cells, low immunogenicity, and high proliferation rate [7]. They secrete growth factors providing a cell therapy potential in constructive surgery [12, 13]. All these properties make ADSCs promising fillers for the conduits in nerve tabulation.

The application of stem cells from any sources is connected with certain risks and methodological difficulties: provocation of oncological diseases [14], low possibility of using autologous cell in emergency operations due to their long-tern cultivation and determination, for example, under military field conditions; legislative restriction of their clinical application in some countries. These difficulties determine the necessity of further search for optimal ways of using stem cells in regenerative medicine. Transplantation of the autologous adipose tissue (AAT) containing native stem cells, which may potentially influence the regenerating nerve fibers (NF), seems to be a perspective solution of this problem [15, 16].

**The aim of the investigation** is to study structural alterations of autologous omental adipose tissue in the silicon conduit and to evaluate its possible use for regeneration of the sciatic nerve in diastasis.

## Materials and Methods

Outbred male Wistar rats 6 months of age weighing 350–450 g have been used in the study. The animals were housed under the standard conditions of vivarium. The work complied with the ethical principles of the European Convention for the Protection of Vertebrate Animals used for Experimental and other Scientific Purposes (Strasbourg, 2006).

The experiments were carried out using isoflurane, Zoomed MinorVet anesthesia apparatus (ZOOMED, Russia), and Armed 7F-3L oxygen concentrator (ARMED, Russia). Animals were divided into six experimental groups and a control group (n=4 in each).

The rats underwent complete transection of the sciatic nerve from the right side at the mid-third level of the thigh. The ends of the nerve were separated, inserted into the silicon conduit (tube) preventing the formation of the connective tissue scar, and secured to the epineurium with 8/0 suture material.

Two types of tubes 10-mm long were used in the experiment: having a complex (type 1) and simple (type 2) construction. The type 1 conduit consisted of two parts with a 2-mm inner diameter and a central part with a diameter of 3 mm, which allowed for the increase of the AAT filler volume. The type 2 conduit represented a one-piece tube with a 2-mm inner diameter.

In animals of *group 1*, the ends of the transected nerve were inserted into the type 1 conduit in such a way that there was a fixed gap of 5 mm between them. The conduit was filled with a saline solution. A follow-up period was 14 weeks.

In *group 2*, the abdominal cavities of the animals were opened to collect the omental tissue (1 ml), then the abdominal muscles and skin were sutured. The omental tissue was further milled in the saline solution and (without any chemical treatment) introduced by a syringe into the type 1 conduit as in group 1. The postoperative follow-up period was 4 and 14 weeks.

In *group 3*, intravital labeling of the omental adipose tissue with the PKH 26 lipophilic dye (Sigma-Aldrich, USA) was used for the first time to find out whether the AAT cells were involved in the formation of the regenerating nerve. This dye was fixed on the cellular membrane and transmitted in the uniform proportion to the daughter cells in mitosis [17]. The PKH 26 is known to remain after transplantation of mature adipose cells for 14.5 months [18]. A known method of labeling multipotent stem cell suspension of the red bone marrow [19, 20] was used in our modification in this group: without chemical pretreatment of the omental AAT and without isolation of the regional stem cells from it.

The milled omental tissue (1 ml) was washed in the saline solution in the centrifuge at 1500 rpm (3 times for 5 min). After removing the supernatant, 1 ml of Dilutent C (Sigma-Aldrich, USA) was added to the sediment. Then, it was stained with the PKH26GL-1KT dye (Sigma-Aldrich, USA) (4 μl of PKH 26: 1 ml of Diluent C, 5-min incubation). Staining was stopped with 1% solution of bovine serum albumin (BSA) in the saline solution. The stained sediment was washed with the saline solution 3 times for 10 min at 1500 rpm. The ends of the transected sciatic nerve were spread 5 mm apart and inserted into the type 1 conduit prefilled with AAT stained with PKH 26. The postoperative follow-up period was 14 weeks. 15-μm thick frozen sections were prepared from the regenerating nerve located in the conduit using Leica CM 1900 UV microtome (Leica Microsystems, Austria) for the fluorescence analysis. The sections were photographed on the LSM 710 confocal laser scanning microscope (Carl Zeiss, Germany) and analyzed.

In *groups 4–7*, the milled omental tissue (without chemical pretreatment) was placed into the type 2 conduit covering 2 mm of diastasis. The postoperative follow-up period in group 4 was 4 weeks, in group 5 — 14 weeks, in group 6 — 21 weeks, in group 7 — 42 weeks.

The forming nerve was taken for the light and transmission electron microscopy at the site of its diastasis in the animals of all groups except for group 3. The material was fixed in the 2.5% glutaraldehyde solution on phosphate-buffer saline (PBS) (рН 7.4) and postfixed in 1% osmium tetroxide solution with subsequent embedding into the epon-araldite mixture following the standard protocol. The morphological analysis was performed on the semi-thin (0.5 μm) sections were obtained using Leica UC7 ultramicrotome (Leica Microsystems, Austria) and stained with methylene blue and fuchsine, and also on the ultrathin sections (70 nm) stained with uranyl acetate and zinc citrate according to Raynolds. Photorecording was done on the Eclipse 80i light microscope with DS-Fi1 camera (Nikon, Japan) with 10x ocular lenses and 10x and 20x objective lenses. The morphological analysis of the cross-sections was performed in the NIS Elements BR 4.0 program (Nikon, Japan). Ultrathin sections were analyzed on the Morgagni 268D transmission electron microscope (FEI, USA).

The total number of the regenerated NFs and percentage of different size groups: small (<4 μm in diameter), middle-sized (4–7 μm), large (>7 μm) were counted for groups 1 and 2.

**Statistical methods**. The qualitative analysis of the nerve fibers of various calibers was performed in experimental groups 1 and 2 using Statistiсa 10.0 program. Non-parametric Mann–Whitney test has been applied, values were considered statistically significant at p<0.025.

## Results

### Clinical assessment of the injured limb condition

**Figure 1.**
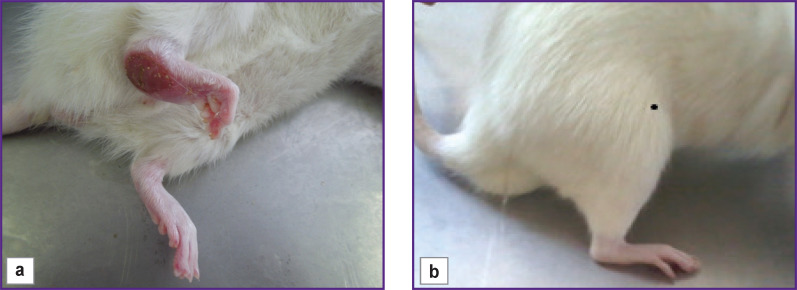
Limb appearance 14 weeks after tabulation of the sciatic nerve: (a) group 1 (conduit is filled with saline solution); (b) group 2 (conduit is filled with autologous omental adipose tissue)

14 weeks after the sciatic nerve transection, the limb of the animals in group 1 looked as follows: a large trophic ulcer was observed on the heel, the ankle joint was greatly thickened, extended over 90–100° (normally, 180°), the toes were partially or completely gnawed off, the rest were bent; when walking, the animals poorly leaned on the injured limb (Figure 1 (a)).

The clinical results obtained have shown that the application of the silicon conduit filled with a saline solution does not lead to proper limb restoration.

In group 2, the animals leaned well on the operated paw when walking, the toes were not curled, straitened when walking. In the ankle, the paw extension angle was 150–160°, the joint was not thickened (Figure 1 (b)). After transplantation of the omental AAT in the diastasis between the stumps, a newly formed nerve region was anatomically significantly thicker than that in the conditionally empty conduit.

### Morphological analysis of the nerve at the conduit site

**Figure 2.**
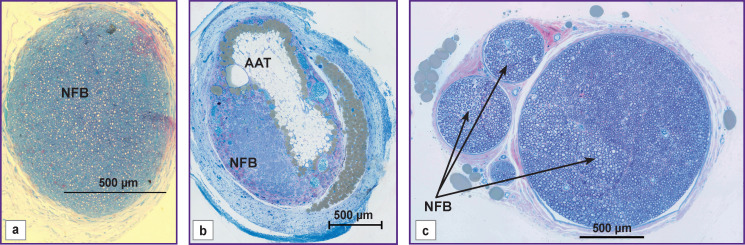
Cross-sections of the sciatic nerve at the silicon conduit site 14 weeks after the operation: (a) group 1; (b) group 2; (c) intact nerve [21]. NFB — nerve fiber bundle, AAT — autologous adipose tissue of the omentum

The analysis of the sciatic nerve section 14 weeks after the experiment has shown that in *group 1* there was one bundle of NFs as opposed to the normal nerve structure [21]. Epineurium, perineurium, and endoneurium were well-defined (Figure 2 (a)). Small NFs prevailed and made up 71.7%, medium-sized fibers — 26.6%, large — 1.7%, respectively, of the total NF number in the given group (see the Table). In this group, blood vessels in the nerve sheaths of the small diameter were encountered rarely. The total scar in the trauma area was not observed. The clinical picture and morphological parameters gave evidence that the predominant small fibers in group 1 do not allow the nerve to function properly. At the same time, absence of the total scar in the diastasis region appeared to be a positive effect of tabulation: it prevented the connective tissue elements from penetration into the trauma zone.

**Table:** The number of myelinated nerve fibers in the sciatic nerve in the experimental groups 14 weeks after the operation (%)

Size groups of myelinated nerve fibers	Group 1	Group 2
Large (d>7 μm)	108	1336*
Medium (4<d<7 μm)	1690	3249*
Small (d<4 μm)	4559	1512*
Total number	6357	6097

Note: mean values across the group are given; * statistically significant differences between the groups, р≤0.025.

**Figure 3.**
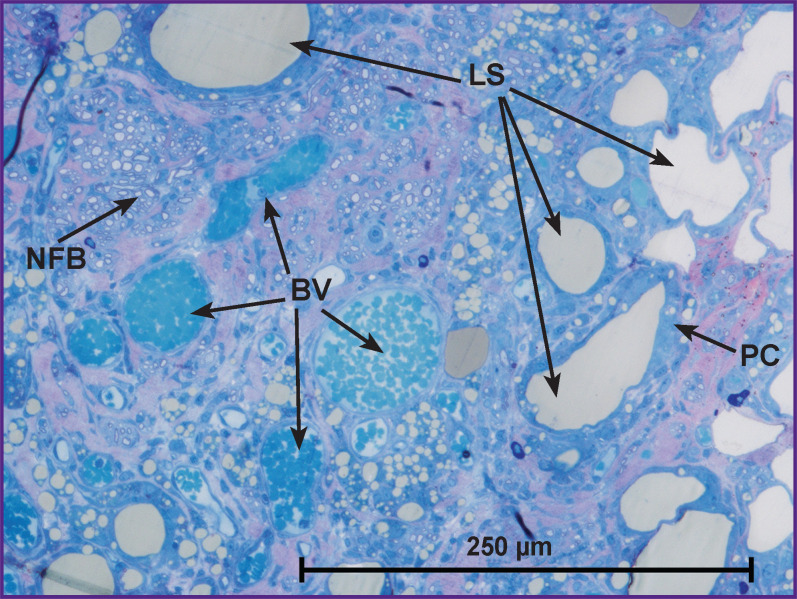
Nerve structure at the conduit site in group 2 after 14 weeks of the experiment LS — lipid structures, BV — blood vessels, NFB — nerve fiber bundle, PC — phagocyting cells on the periphery of LS; oc. — ×15; ob. — ×20

In *group 2*, the morphological analysis has shown that the cross-section area of the nerve significantly exceeded this value in group 1 (Figure 2 (b)). All sheathes were well defined. Epineurium was more developed. In the trunk, the predominance of the large and medium-sized NFs was found as compared with the values for group 1: medium-sized — 53%, large — 22%, and small — 25% (of the total NF number in the given group) (see the Table). Vascularization was enhanced in comparison with group 1 (Figure 3). In this group, the predominance of the medium-sized NFs may provide functioning of the nerve in contrast with the small fibers. This fact has been noted in our previous study [21].

It is known that ADSCs, isolated from the subcutaneous and interstitial fat, are able to integrate into the regenerating nerve, be directly involved in its reconstruction, and increase expression of some neurotrophic factors [16, 22–24].

The clinical state of the injured animal limb in group 2 has shown a graft from the AAT being a kind of “armature” guiding and facilitating regeneration of the sciatic nerve.

In group 2, the regenerated nerve had a heterogeneous structure: the connective tissue stroma with areas of fibrosis and lipid structures was pierced with blood vessels and NF bundles (see Figure 2 (b), Figure 3). Similar results were also observed by other authors after transplantation of the adipose tissue [16, 25], when scars and formations which they called lipid cysts were formed after degeneration and necrosis. Phagocytosis of adipocytes by macrophages and their resorption took from several weeks to months [26]. According to our data, after 14 weeks, the graft lipids were not fully utilized, and the process continued.

**Figure 4.**
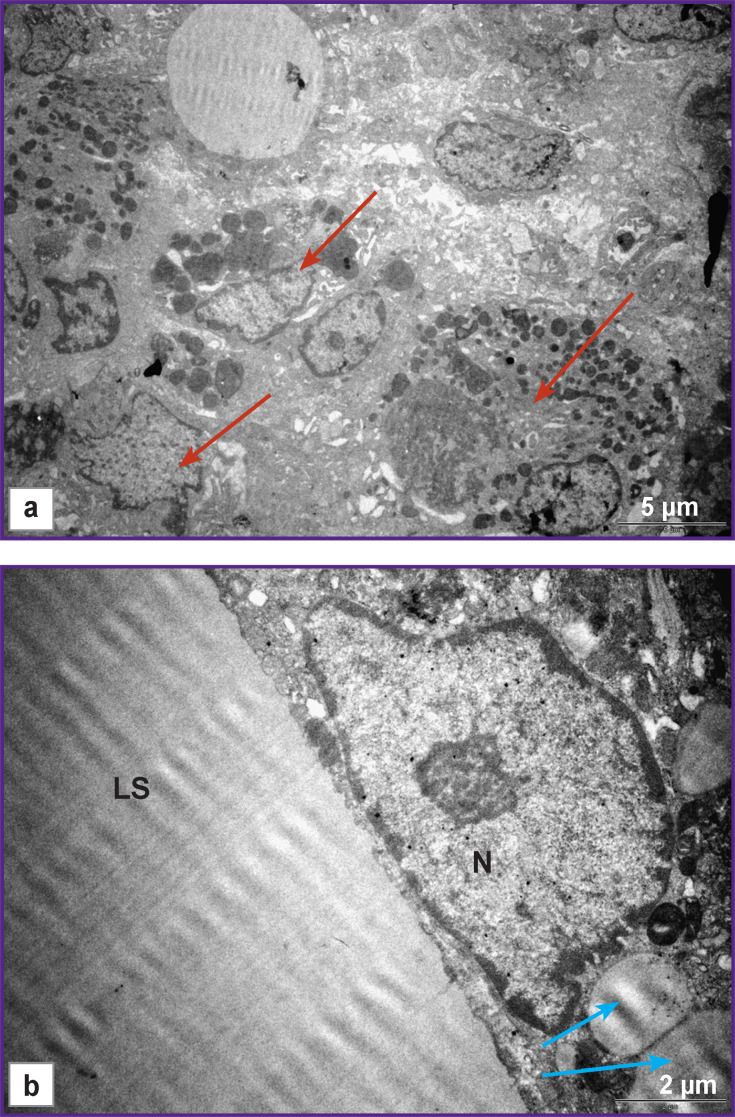
Nerve section at the conduit site in group 2: (a) 4 weeks of the experiment, phagocyting cells are shown by red arrows; (b) 14 weeks of the experiment, a phagocyting cell. LS — lipid structure, N— nucleus of the phagocyting cell, lipid inclusions are shown by blue arrows

In this period (14 weeks), lipid structures different in shape and size were surrounded with the cells of unidentified phenotype tightly fitting around the periphery and participating in their utilization. At the ultrastructural level, the cells had a diverse form, large bright nuclei with a nucleolus were noted, widening of the smooth endoplasmic reticulum cisternae was observed, the cytoplasm was filled with lipid inclusions. The signs of active phagocytosis appeared in 4 weeks (Figure 4 (a)). According to the data of some authors [27, 28], Schwann and perineural cells utilized myelin in Wallerian and retrograde degeneration of the damaged peripheral NFs. The probability of involvement of these cells in phagocytosis of the lipid structures in our experiment cannot be excluded.

**Figure 5.**
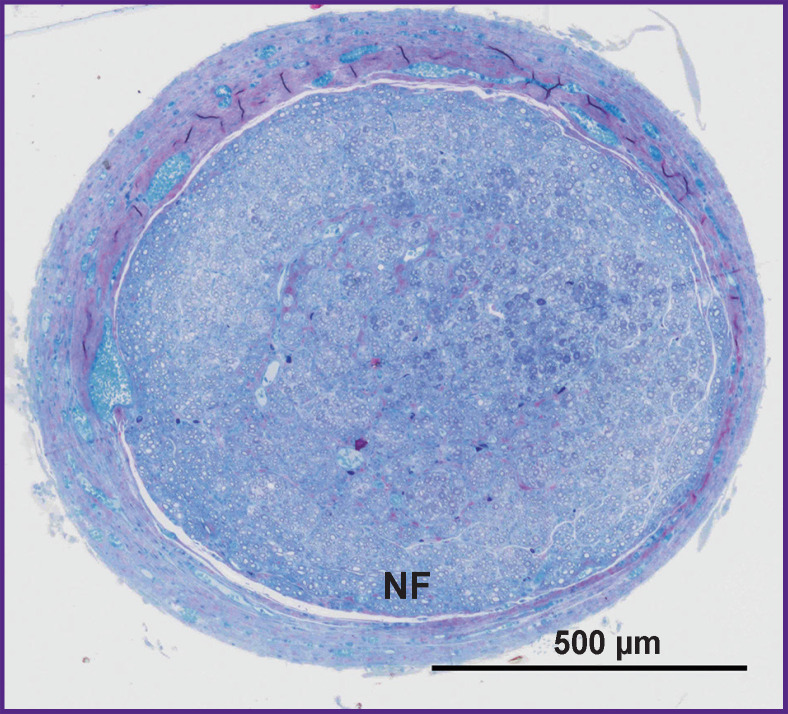
Cross-section of the nerve at the conduit site after 42 weeks (group 7) NF — nerve fibers

In *groups 4–7*, gradual utilization of the lipid structures was also going on. Four weeks after the transplantation, lipid conglomerates in the sections prevailed, NFs were single. After 21 weeks of the experiment (group 6), AAT was still present in small quantities, by week 42 (group 7) it was completely utilized, the regenerated nerve was filled with NF bundles (Figure 5).

In the experiment [29], milled adipose tissue from the back area placed in the diastasis to 1 cm of the median nerve length did not have any positive effect on its regeneration after 6 months. The authors believed that a large amount of unutilized lipid conglomerates had blocked the NFs growth. It is likely to be connected with a large diastasis size.

**Figure 6.**
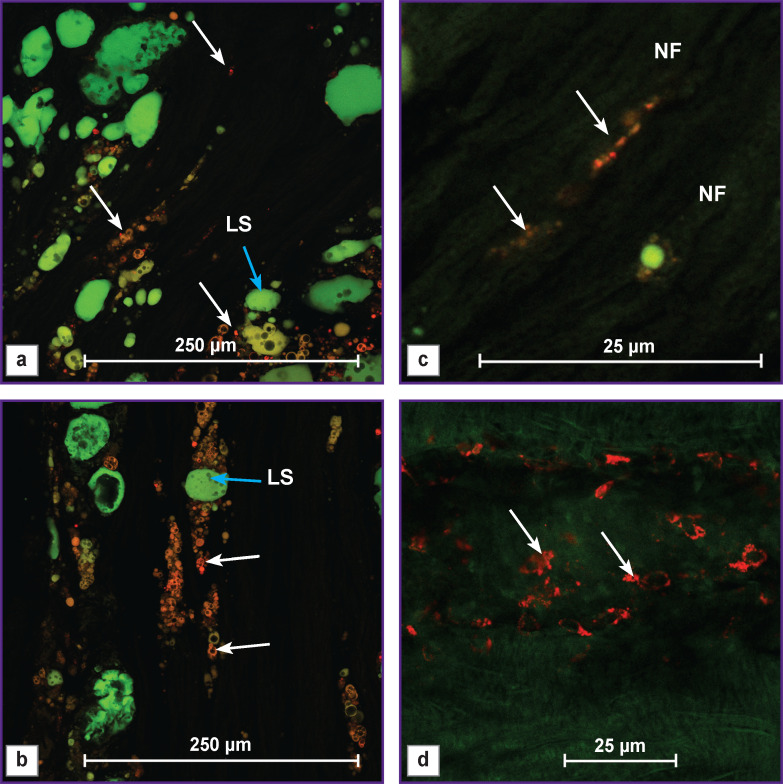
Longitudinal section of the nerve at the conduit site (group 3) after 14 weeks of the experiment White arrows show the regions containing cells with red fluorescence positive for PKH 26 stain which are located near LS (a), (b), along NF (c), and in the epineurium (d). LS — lipid structures, NF — nerve fibers

In *group 3* with the intravital labeling of the omental adipose tissue with the PKH 26 stain, cells with red fluorescence were observed. They were distributed in three zones: near lipid structures (Figure 6 (a), (b)), along NFs (Figure 6 (c)), and in the epineurium (Figure 6 (d)).

In Figure 6 (a), (b), small red cells with dark rounded cavities of different sizes were located near lipid structures with green endogenous fluorescence. Similar morphological picture was observed by the authors in the study [22] investigating cultured ADSCs stained with Oil Red O, who explained this phenomenon by adipogenetic differential induction in the stem cells. Therefore in our study, part of ADSCs of the transplanted omentum might differentiate into new lipid cells.

The question of adipose cell survival after milling the omental tissue in our experiment remains open. Some authors [16, 26, 30, 31] believe that an essential part of adipocytes in the lipid graft is subject to necrosis during 24 h after the operation. In our experiment, the lipid cells encountered after 14 weeks are supposed to be newly formed.

In group 3, we found the cells with red fluorescence along the longitudinally cut NFs with green endogenous fluorescence. The final phenotype of these cells was not established in our work, however, it was seen on the preparations that the РКН 26-labelled omental cells had integrated into the newly formed nerve area (see Figure 6 (c)). Similar results have been also obtained by other authors in the investigations with cultivated and non-cultivated ADSCs.

In their study [22], the authors placed ADSCs of the Wistar rats with acellular allograft in the diastasis of the transected sciatic nerve. Twelve weeks later, they identified the РКН 26-labeled cells with red fluorescence and elevated expression of the neurotrophic factors BDNF, NT-3, and GDNF, which spoke of a long-term survival of these cells and their stimulating effect on nerve regeneration. It should be noted that identification of ADSCs was not conducted by the authors.

Cultivated human ADSCs prelabeled with PKH 26 were transplanted into the biodegradable polycaprolactone conduit covering the 6 mm of diastasis in the injured rat sciatic nerve [32]. After 12 weeks, the PKH 26-labeled cells with red fluorescence without colocalization with NF neutrophin identifying the growing axons were observed in the graft area. The authors have made the conclusion that the stem cells did not differentiate into Schwann cells. It might be connected with the inability to stain each cell in the section, and the authors admitted the possibility of such differentiation.

In the study [24], the 10 mm of diastasis on the peripheral nerve was covered with a silicon conduit filled with a mixture of non-cultivated ADSCs and type I collagen. The degree of regeneration in this group was evaluated as satisfactory. At the same time, the distribution of regions positive for PKH 26 and S100 protein was different. Therefore, the authors have assumed that transplanted non-cultivated ADSCs do not differentiate into Schwann cells but integrate into the regenerated nerve and participate in the expression of neuruline-1 and vascular endothelial growth factor A, which promote proliferation or migration of the Schwann cells.

Analyzing the literature data, it may be concluded that adipose semi-stem cells integrate into the newly generated nerve at the trauma site, but the phenotype of these cells is not established.

In our experiment, cells with red fluorescence containing PKH 26 and having a dendritic shape were also observed in the epineurium area. It is likely that part of ADSCs has transformed into fibroblasts. Similar phenomenon was also shown by the authors [22] in the ADSC culture.

The analysis of the data obtained by us gives grounds to assume that the stem cells from the milled omental AAT migrate in certain zones of the generating nerve, incorporate into it, and promote its reconstruction.

## Conclusion

Autologous adipose tissue of the milled omentum transplanted inside the silicone conduit in the transected sciatic nerve diastasis changes dynamically in its volume and structure undergoing constant and considerable resorption and utilization. As a result, there is a tendency to its complete disappearance over time and replacement with regenerated nerve fibers. In the process of structural changes, the adipose tissue is, on the one hand, a mechanical barrier for the growing nerve fibers at the early stages, while on the other, it produces a stimulating effect on the sciatic nerve regeneration and restoration of the damaged limb functions.

The results of this study give grounds for further exploration of the mechanisms by which the autologous omental adipose tissue influences post-traumatic regeneration of the peripheral nerve.
